# Genotyping of extended-spectrum beta-lactamase-producing *Escherichia coli* isolated from wastewater of dairy farms in East Java, Indonesia

**DOI:** 10.14202/vetworld.2025.162-171

**Published:** 2025-01-27

**Authors:** Fidi Nur Aini Eka Puji Dameanti, Sheila Marty Yanestria, Mustofa Helmi Effendi, Hani Plumeriastuti, Wiwiek Tyasningsih, Emmanuel Nnabuike Ugbo, Rahayu Sutrisno, Muhammad Ali Akramsyah Safri

**Affiliations:** 1 Laboratory of Microbiology and Immunology Veterinary, Faculty of Veterinary Medicine, Universitas Brawijaya. Jl. Puncak Dieng, Kalisongo, Malang Regency, 65151. East Java, Indonesia; 2 Department of Veterinary Public Health, Wijaya Kusuma Surabaya University, Jl. Dukuh Kupang XXV No.54, Dukuh Kupang, Dukuhpakis, Surabaya, 60225, East Java, Indonesia; 3 Department of Veterinary Public Health, Faculty of Veterinary Medicine, Universitas Airlangga, Jl. Dr. Ir. H. Soekarno, Kampus C Mulyorejo, Surabaya, 60115, East Java, Indonesia; 4 Department of Veterinary Pathology, Faculty of Veterinary Medicine, Universitas Airlangga, Jl. Dr. Ir. H. Soekarno, Kampus C Mulyorejo, Surabaya, 60115, East Java, Indonesia; 5 Department of Veterinary Microbiology, Faculty of Veterinary Medicine, Universitas Airlangga, Jl. Dr. Ir. H. Soekarno, Kampus C Mulyorejo, Surabaya, 60115, East Java, Indonesia; 6 Department of Applied Microbiology, Faculty of Science, Ebonyi State University. 480211, Abakaliki, Nigeria; 7 Department of Animal Health, Unggas Karya Mandiri, Malang Regency 65151. East Java, Indonesia

**Keywords:** antibiotic resistance, *bla*_CTX-M_, *bla*_SHV_, *bla*_TEM_, dairy farm wastewater, ESBL-producing *Escherichia coli*, Indonesia, public health

## Abstract

**Background and Aim:**

Extended-spectrum beta-lactamase (ESBL)-producing *Escherichia coli* in dairy farm wastewater represents a significant threat to environmental and public health due to the dissemination of antibiotic-resistance genes. This study investigates the molecular profiles of ESBL-producing *E. coli* isolates harboring *bla*_CTX-M_, *bla*_TEM_, and *bla*_SHV_ genes from dairy farm wastewater in East Java, Indonesia.

**Materials and Methods:**

Wastewater samples (n = 342) were collected from six major dairy regions in East Java: Pasuruan, Malang, Tulungagung, Blitar, Batu, and Kediri. The samples underwent bacterial isolation, identification, and ESBL phenotypic screening using the double-disk synergy test (DDST). Molecular genotyping of *bla*_CTX-M_, *bla*_TEM_, and *bla*_SHV_ was conducted using a polymerase chain reaction.

**Results:**

Of the samples, 69.30% (237/342) tested positive for *E. coli*, with 32.91% (78/237) identified as ESBL producers through DDST. Molecular genotyping confirmed ESBL genes in 28.20% (22/78) of the isolates. The genotypic distribution was 77.2% (17/22) *bla*_TEM_, 4.5% (1/22) *bla*_CTX-M_, 9.0% (2/22) *bla*_TEM_ + *bla*_SHV_, and 9.0% (2/22) *bla*_TEM_ + *bla*_CTX-M_. These findings highlight the dominance of *bla*_TEM_ and the presence of multi-gene combinations in East Java’s dairy farm wastewater.

**Conclusion:**

The identified molecular profiles underscore dairy farm wastewater as a critical reservoir for antibiotic resistance genes, particularly *bla*_TEM_. Addressing this issue necessitates stringent antimicrobial use policies, improved wastewater management, and enhanced biosecurity measures. These findings support a One Health approach to mitigate environmental and public health risks associated with AMR.

## INTRODUCTION

Antimicrobial resistance (AMR) is a serious problem because it affects the three aspects of “One Health”, namely humans, animals, and the environment [1]. As an intermediary for humans and animals, the environment is a source of acquisition and horizontal gene transfer of antibiotic resistance; thus, the environment is considered the main route of transmission of resistant genes by bacteria to the community [2, 3]. The environment, waste management, and feed used in dairy farming influence the development and spread of AMR [4]. Banu *et al*. [2] have explained that AMR problems and environmental pollution are largely caused by meeting high food needs with low knowledge gaps in communities. Dairy farming in Indonesia is dominated by traditional farms, which causes various problems. The lack of rational use of antibiotics and disposal of untreated waste cause environmental pollution around farms [5, 6]. Resistant bacteria in livestock can migrate around farms through water sources, irrigation, rivers, rainwater, and polluted food chains, affecting the environmental microbiota and humans [7–9]. Livestock wastewater has become the main storehouse for antibiotics and other resistant bacteria [10].

The increasing incidence of AMR worldwide, particularly extended-spectrum beta-lactamase (ESBL)-producing strains of *Escherichia coli*, is causing serious health problems. ESBL can hydrolyze penicillin and cephalosporin antibiotics [11]. ESBL enzymes have become a worldwide concern because genes encoding these genes are mainly on plasmids that can also carry many other AMR genes (ARGs). Previous studies indicate that ESBL-producing bacteria exhibit multidrug resistance (MDR) phenotypes and carry multiple resistance genes encoding resistance to aminoglycosides, chloramphenicol, sulfonamides, tetracyclines, and macrolides [6, 12–14]. The ESBL gene on the plasmid is located in the mobile genetic element (MGE) and can be easily transmitted to other bacteria, thereby increasing the prevalence of ESBL [15, 16]. The results showed that breeders and cattle from Europe had *E. coli* ESBL plasmids that were related and identical [17]. Poor hygiene increases the risk of this incident on dairy farms in Indonesia. ESBL-producing *E. coli* is not only found in dairy cattle but also in various sources, such as the feces of residents around the farms in Surabaya; wastewater, feed, farmer’s hand rinsing, and animal drinking water in Sleman, Yogyakarta; thus, the environment plays an important role in increasing the prevalence of ESBL [10, 18].

The dairy farm environment has a high potential to become a reservoir for spreading ESBL-producing *E. coli*. The ESBL variants produced by *E. coli* belong to the TEM, SHV, and CTX-M enzyme groups encoded by *bla*_CTX-M_, *bla*_TEM_, and *bla*_SHV_ genes, respectively [19]. Since the first ESBL TEM-1 was discovered in 1965 in *E. coli* isolates, these three variants have developed and continued to mutate [14, 20]. ESBL gene mutations lead to increased transmission of antibiotic-resistance genes among breeders, animals, and animal products [21–23]. Therefore, it is important to identify molecular characteristics of ESBL-producing *E. coli* from dairy farm wastewater.

This study aimed to determine the potential occurrence and molecular characteristic profiles of the *bla*_CTX-M_, *bla*_TEM,_ and *bla*_SHV_ genes of ESBL-producing *E. coli* isolates from dairy farm wastewater in East Java.

## MATERIALS AND METHODS

### Ethical approval and informed consent

Ethics approval was not required for this study as it did not involve live animals. The owners have provided their verbal informed consent and signed the consent form to participate in this study.

### Study period and location

This study was conducted from June 2022 to June 2023. Samples were collected from 342 dairy farms in six cities/regencies with the highest dairy cattle population in East Java Province: Pasuruan, Malang, Tulungagung, Blitar, Batu, and Kediri Regencies. The isolation, identification, and phenotyping were performed at the Laboratory of Microbiology and Immunology Veterinary, Faculty of Veterinary Medicine, Universitas Brawijaya, Indonesia. PCR molecular testing was performed at the Institute of Tropical Disease Center, Universitas Airlangga, Indonesia.

### Sample collection, isolation, and identification of *E. coli*

Wastewater samples (each 100 mL) were collected from 342 ditches of dairy farms aseptically and stored in centrifuge tubes. The samples were stored in a cool box and transported to the laboratory for analysis within 24 h [10]. Specifically, 5 mL of wastewater samples were isolated in 2% Buffer Peptone Water Media (Oxoid, UK) with a 1:1 ratio as the pre-enrichment medium and incubated at 37°C for 24 h [24]. Samples were continued on eosin methylene blue agar (EMBA; Oxoid) enrichment media by streaking using round loops in 4 quadrants and incubating at 37°C for 24 h. Colonies that appeared metallic green were continued on Gram staining to determine the morphology and properties of the bacteria [25]. The isolates were subjected to biochemical tests using the IMViC test (Indole-motility [HiMedia, India], Methyl Red & Voges Proskauer [HiMedia], Citrate [HiMedia]), Triple Sugar Iron Agar (TSIA) (HiMedia), and Urease (HiMedia) to identify *E. coli* isolates. The isolation and identification results were performed based on a previous study by Dameanti *et al*. [5].

### ESBL phenotyping

ESBL-producing *E. coli* isolates were confirmed using the double-disk synergy test (DDST) based on standards from the Clinical and Laboratory Standards Institute [26]. DDST was performed using the Kirby–Bauer disk diffusion method on Mueller–Hinton Agar (MHA; Oxoid) media with three types of antibiotics: Ceftazidime (CAZ, 30 g; Oxoid), cefotaxime (CTX, 30 g; Oxoid), and amoxicillin-clavulanic (AMC, 30/10 μg; Oxoid) [27]. *E. coli* isolates suspended according to the McFarland 0.5 standard were swabbed on MHA media [28]. After 15 min, the antibiotic disks were placed on the surface of the media 15 mm apart and incubated at 37°C for 24 h. Samples confirmed the ESBL phenotype when an inhibition zone diameter ≥5 mm between the cephalosporin inhibition zone (cefotaxime and ceftazidime) and amoxicillin-clavulanic acid was observed [5, 29]. The increase in the zone is caused by clavulanate on the antibiotic disk, which deactivates ESBL produced by bacteria [30].

### Genotyping of ESBL-producing *E. coli*

Isolates that were phenotypically confirmed as ESBL producers were continued with the polymerase chain reaction (PCR) amplification test to identify the presence of *bla*_CTX-M_, *bla*_TEM_, and *bla*_SHV_. *E. coli* isolates from the DDST test were re-cultured on Nutrient Agar (NA; Oxoid) medium and incubated at 37°C for 18–24 h. Several colonies were then taken using a sterile inoculation loop and transferred to an Eppendorf tube filled with 100 μL of sterile aquadest and 5 μL of lysozyme and then incubated at 100°C for 5 min to lyse the cells. The samples were then centrifuged at 8.944x *g* for 5 min at 4°C. Next, the sample DNA pellet/supernatant was diluted to 100 μl with a buffer kit (QiaAmp DNA Mini Kit 50) [23, 31]. The primers for each gene were obtained from the literature [32]. The primary details of each gene and the PCR amplification process are presented in Table 1 [23, 33]. PCR results were visualized by electrophoresis using 2% agarose gel (Invitrogen, USA), stained with ethidium bromide, and visualized using ultraviolet light [31, 33].

**Table 1:** Primers of ESBL genes.

Gene	Primary sequence (5’→3’)	PCR conditions	Application size (bp)
*bla*_TEM_	F: 5’ ATGAGTATTCAACATTTCCG 3’	1 cycle of 5 min at 96°C; 35 cycles of 1 min at 96°C; 1 min at 58°C; 1 min at 72°C; 1 cycle of 10 min at 72°C	867
	R: 5’ CTGACAGTTACCAATGCTTA 3’		
*bla*_SHV_	F: 5’ GGTTATGCGTTATATTCGCC 3’	1 cycle of 5 min at 96°C; 35 cycles of 1 min at 96°C; 1 min at 60°C; 1 min at 72°C; 1 cycle of 10 min at 72°C	867
	R: 5’ TTAGCGTTGCCAGTGCTC 3’		
*bla*_CTX-M_	F: 5’ ATGTGCAGYACCAGTAARGT 3’	1 cycle of 7 min at 94°C; 35 cycles of 50 s. at 94°C; 40 s. at 50°C; 1 min at 72°C; 1 cycle of 10 min at 72°C	593
	R: 5’ TGGGTRAARTARGTSACCAGA 3’		

bp=Base pair, PCR=Polymerase chain reaction, ESBL=Extended-spectrum beta lactamase

## RESULTS

### Isolation and identification of *E. coli*

The results of the isolation and identification of *E. coli* from 342 samples of dairy farm wastewater were positive for *E. coli* at 69.30% (237/342). These results are distributed across the cities/regencies as follows: 19.41% (46/237) in Kediri, 13.08% (31/237) in Blitar, 18.57% (44/237) in Malang, 18.14% (43/237) in Batu, 19.41% (46/237) in Pasuruan, and 11.39% (27/237) in Tulungagung.

### Phenotypic screening for ESBL production

A total of 32.91% (78/237) of isolates were found to be ESBL producers. Details of the screening results for each city/regency include 14.10% (11/78) from Kediri, 15.54% (12/78) from Blitar, 11.54% (9/78) from Malang, 20.51% (16/78) from Batu, 20.51% (16/78) from Pasuruan, and 17.95% (14/78) from Tulungagung. The *E. coli* DDST results are shown in Table 2.

**Table 2:** Isolation and identification results; DDST and PCR tests of ESBL-producing *Escherichia coli* isolates.

City/District	Isolation and identification		Confirm ESBL with the DDST		ESBL confirmation by PCR						
		
	n	%	n	%	N	CTX-M	%	SHV	%	TEM	%
Kediri	46	19.41	11	14.10	5	0	0	0	0	5	23.8
Blitar	31	13.08	12	15.54	1	0	0	0	0	1	4.8
Malang	44	18.57	9	11.54	2	1	33	0	0	1	4.8
Batu City	43	18.14	16	20.51	7	1	33	1	50	7	33.3
Pasuruan	46	19.41	16	20.51	4	1	33	0	0	4	19.0
Tulungagung	27	11.39	14	17.95	3	0	0	1	50	3	14.3
Total	237	100	78	100	22	3	100	2	100	21	100

ESBL=Extended-spectrum beta-lactamase, DDST=Double disk synergy test, PCR=Polymerase chain reaction

### Genotyping of ESBL-producing *E. coli*

**Figure 1:**
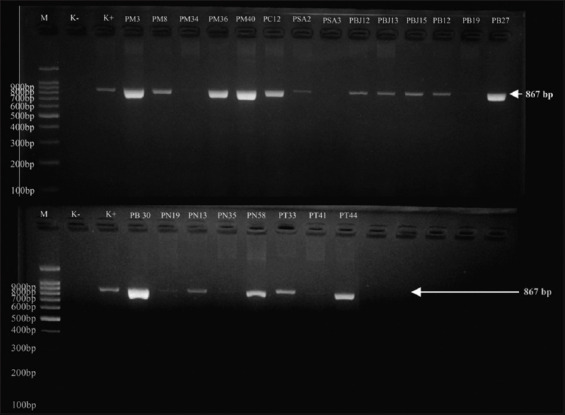
Polymerase chain reaction electrophoresis results with visualization of the TEM (Temoneira) gene fragment band (867 bp). M=Marker, K-=Negative control, K+=Positive control, PM=Kediri Regency sample, PC=Malang Regency sample, PSA=Blitar Regency sample, PB/PBJ=Batu City sample, PN=Pasuruan Regency sample, PT=Tulungagung Regency sample.

**Figure 2:**
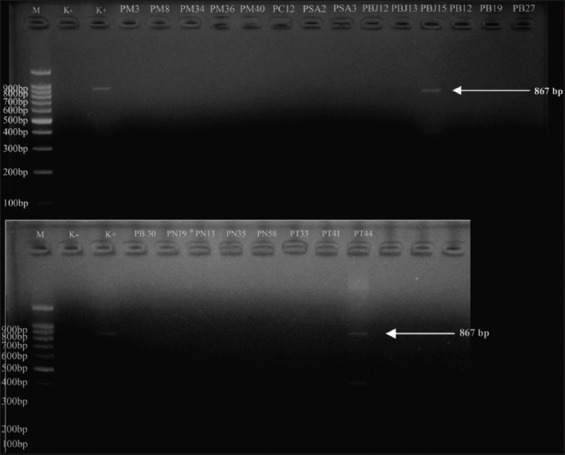
Polymerase chain reaction electrophoresis results with visualization of the SHV gene fragment band (867 bp). M=Marker, K–=Negative control, K+=Positive control, PM=Kediri Regency sample, PC=Malang Regency sample, PSA=Blitar Regency sample, PB/PBJ=Batu City sample, PN=Pasuruan Regency sample, PT=Tulungagung Regency sample.

**Figure 3:**
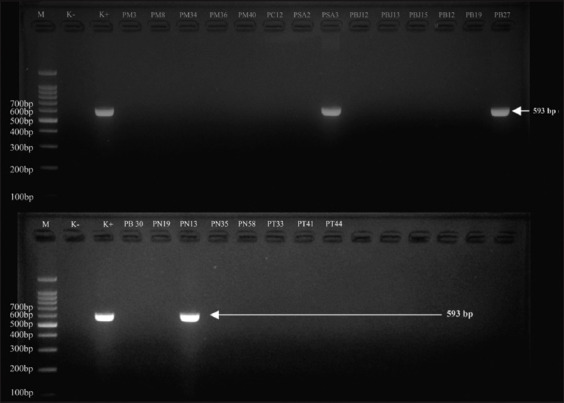
Polymerase chain reaction electrophoresis results with visualization of the CTX-M gene fragment band (593 bp). M=Marker, K-=Negative control, K+=Positive control, PM=Kediri Regency sample, PC=Malang Regency sample, PSA=Blitar Regency sample, PB/PBJ=Batu City sample, PN=Pasuruan Regency sample, PT=Tulungagung Regency sample.

All *E. coli* isolates that were positive for DDST were then genotypically characterized by *bla*_CTX-M_, *bla*_TEM,_ and *bla*_SHV_ using PCR. The PCR test results were positive for 28.20% (22/78) of *E. coli* ESBL. The PCR results are detailed in Table 2. The ESBL genotypes were positive for 77.2% (17/22) *bla*_TEM_, 4.5% (1/22) *bla*_CTX-M_, 9.0% (2/22) *bla*_TEM_+*bla*_SHV_, and 9.0% (2/22) *bla*_TEM_ + *bla*_CTX-M_ as shown in Table 3. PCR molecular identification showed visualization of the *bla*_TEM_ gene fragment band at 867 bp, as shown in Figure 1; the *bla*_SHV_ gene fragment band at 867 bp, as shown in Figure 2; and the *bla*_CTX-M_ gene fragment band at 593 bp, as shown in Figure 3. The negative control in this test was *E. coli* ATCC 25922, whereas the positive control was *Klebsiella pneumoniae* ATCC 700603. This strain was selected by the National Committee for Clinical Laboratory Standards as a quality control to improve the detection of ESBLs in *Enterobacteriaceae* in DDST and PCR testing [34].

**Table 3:** Combinations of ESBL-producing *Escherichia coli* genotypes.

ESBL genotype	Number of isolates	

	N	%
TEM	17	77.2
CTX-M	1	4.5
TEM+SHV	2	9
TEM+CTX-M	2	9
Total	22	100

ESBL=Extended-spectrum beta lactamase

## DISCUSSION

The results showed that the wastewater samples from dairy farms in East Java were positive for *E. coli*. *E. coli* has been closely related to mastitis in dairy cattle and diarrhea in humans [27, 35]. The findings of the research conducted by Effendi *et al*. [36] indicated that 73.5% of the *E. coli* bacteria present on broiler farms in Blitar, East Java, Indonesia, were pathogenic, as evidenced by the detection of the virulence genes *iss* and *pap*C. These pathogenic bacteria can potentially cause a range of infections, including septicemia, urinary tract infections, and bacteremia. *E. coli* is also often considered a sign of contamination and used as an indicator of antibiotic resistance in the environment and animals [37]. Our results indicate that more *E. coli* in dairy farm wastewater increases the potential risk to public health. ESBL-producing *E. coli* is increasingly detected in humans worldwide [38]. The World Health Organization [39] and Antimicrobial Resistance Collaborators [40] stated that cases of death due to AMR reach 700,000/year and will increase annually to 10 million in 2050. ESBL caused a loss of USD 1.2 billion in US patient care in 2017 [41].

PCR testing and ESBL phenotypic detection showed different results. In general, there are groups of ESBL, such as TEM, SHV, CTX-M, OXA, IBC, PER, BES, GES, SFO, TLA, and VEB [41, 42]. The common ESBL proteins produced by *E. coli* are TEM, SHV, and CTX-M [43]. The advantage of the phenotypic detection method was not only found in *bla*_CTX-M_, *bla*_TEM_, and *bla*_SHV_ but also in other genes. PCR testing can be a gold standard for confirming ESBL [44, 45].

In this study, the PCR molecular genotype profile of *E. coli* isolates showed the dominance of *bla*_TEM_, followed by *bla*_CTX-M_ and *bla*_SHV_. *E. coli* is the primary bacterium responsible for disseminating antibiotic resistance [46]. Studies on ESBL have been reported on various samples in East Java and found *bla*_TEM_ 12%, *bla*_CTX-M_ 72%, and *bla*_SHV_ 0% in dairy cows in Surabaya [22]; *bla*_TEM_ 21.74% (5/23) of pigs in Malang [47]; as well as *bla*_TEM_ 1.7% and *bla*_CTX-M_ 5.2% of dairy cattle in East Java [48], *bla*_CTX-M_ 80%, and *bla*_SHV_ 10% of layer chickens in Blitar [49]. *K. pneumoniae* in wastewater from dairy farms in East Java was also reported as *bla*_SHV_ gene 63.2% (12/19) and *bla*_TEM_, 31.58% (6/19) [50]. Similar studies on *E. coli* isolates from dairy cattle wastewater were carried out in several countries; New Zealand detected *bla*_CTX-M_ at 3.57% (1/28), and Chiang Mai, Thailand, found *bla*_TEM_ 60.15% (80/133), *bla*_CTX-M_ at 100% (133/133) and *bla*_SHV_ 0% [21]. These results demonstrate that the genotype of ESBL-producing *E. coli* is generally different in countries and regions because of different policies on the use of antibiotics [20]. However, previous studies showed that *bla*_CTX-M_ and *bla*_TEM_ are dominant in *E. coli* [22, 23].

Antibiotic resistance in bacteria can arise from two main processes: Spontaneous mutations in chromosomal genes and acquisition of resistance genes through horizontal gene transfer from other bacterial species [51]. Although the likelihood of spontaneous mutation is low, the vast populations of bacteria worldwide, combined with the widespread and uncontrolled use of antibiotics, can cause constant mutations. Antibiotic resistance is often related to genetic mutations encoded on the bacterial chromosome. For instance, the ESBL TEM-1 gene was first identified in 1965 in *E. coli* isolates and is known to undergo a mutation, specifically a substitution mutation in which threonine at residue 182 is replaced by methionine. This gene continues to mutate and generate more than 246 TEM derivatives of lactamase enzymes [14, 20, 52, 53].

The presence of ESBL TEM is associated with the misuse of cefotaxime antibiotics. TEM-1 can hydrolyze the generation of penicillin and early cephalosporins, such as cefazolin and cephaloridine [54]. This is supported by some TEM beta-lactamases that readily hydrolyze cefotaxime [14]. The observed increase in resistance to cefotaxime antibiotics can be attributed to the significant increase in antibiotic usage by the general population during the COVID-19 pandemic in 2020. The data presented by Qibtiya [55] indicated that the utilization of third-generation cephalosporin antibiotics, including ceftriaxone and cefotaxime-sulbactam, is increasing in the treatment of patients infected with COVID-19. This suggests that resistance to cefotaxime in dairy farm wastewater may be acquired from humans and farmers. Another study on the incidence of ESBL in human clinics/hospitals in Nigeria and Iraq showed that the prevalence of *bla*_TEM_ was more dominant than *bla*_CTX-M_ and *bla*_SHV_ [56]. In other countries, the *bla*_TEM_ gene is the most common ESBL-encoding Gene and causes infection in humans and animals [57]. In addition, this study identified genotypic patterns/combinations of *bla*_TEM_ + *bla*_SHV_ and *bla*_TEM_+ *bla*_CTX-M_. This can lead to various problems, especially fatality rates and limited treatment options [58]. The results of the previous study by Poirel *et al*. [59] showed that the combination of CTX-M enzymes with other enzymes decreased the function of meropenem.

The incidence of *E. coli* ESBL, according to the PCR test in this study, was 28.20% (22/78). Although the incidence is smaller than that of Thailand 88.7% (133/150), It must remain a priority for the government because *E. coli* in livestock wastewater can contribute to clonal and horizontal gene transfer of the ESBL gene to other bacteria in the environment [18, 60]. Mobile genetic components (MGEs), including plasmids, insertion elements, bacteriophages, transposons, integrons, and genomic islands, enhance the rapid spread of ESBL genes [61]. These MGEs are transferred between bacteria through conjugation, transposition, natural transformation, and transduction [62]. Conjugation plays a crucial role in these events. ESBLs are often linked to the conjugation process in bacteria. Conjugation is a mechanism by which DNA is transferred from donor bacteria to recipient bacteria, facilitated by pili on the cell surface or adhesins. This process is supported by the conjugative machinery, which is encoded by genes found on autonomously replicating plasmids or integrative conjugative elements within the chromosome. Furthermore, the increase in antibiotic-resistant bacterial populations can resist antibiotics, and these resistant bacteria will frequently engage in horizontal gene transfer with other bacteria [51, 63, 64].

Dairy farm wastewater is a major source of resistant bacteria [65]. Poor hygiene at dairy farms in Indonesia has caused bacteria containing resistant genetic elements to be spread and contaminate the environment around the farm [5]. It has been demonstrated that ESBL-producing *E. coli* in the environment is closely associated with a deficiency in environmental sanitation procedures during the milking process and contamination of raw milk with these bacteria [66]. Furthermore, the prevalence of ESBL-producing *E. coli* considerably impacts human and animal health. The health complications associated with multidrug-resistant bacterial infections may include increased medical expenses, restricted therapeutic alternatives, prolonged hospitalization, and even mortality [67]. Therefore, it is important to increase the application of good biosecurity, water quality, waste treatment, and cage sanitation as risk factors for ESBL cases in dairy farms [68]. There is a high possibility of transmitting ESBL genes from livestock to humans, so the government needs to develop appropriate interventions and strict policies on antimicrobial use and dairy cattle management to reach a healthier community [69, 70].

## CONCLUSION

This study successfully characterized ESBL-producing *E. coli* isolated from dairy farm wastewater in East Java, Indonesia, focusing on the prevalence and molecular profiles of *bla*_CTX-M_, *bla*_TEM_, and *bla*_SHV_ genes. Among the 342 wastewater samples analyzed, 69.30% tested positive for *E. coli*, and 32.91% of these isolates were confirmed as ESBL producers through phenotypic screening. Molecular analysis revealed that 28.20% of the phenotypic ESBL-positive isolates carried ESBL genes, with *bla*_TEM_ being the most prevalent (77.2%), followed by *bla*_CTX-M_ (4.5%) and combinations of *bla*_TEM_ + *bla*_SHV_ (9.0%) and *bla*_TEM_ + *bla*_CTX-M_ (9.0%).

The study’s strengths include comprehensive sampling from six key dairy-producing regions in East Java, dual characterization methods combining phenotypic and genotypic approaches, and its relevance to public health under the One Health framework by highlighting the role of dairy farm wastewater as a reservoir of antibiotic resistance genes.

However, the study is limited by the absence of detailed statistical validation to confirm regional variations and phenotypic-genotypic agreement, the narrow scope of resistance genes analyzed, and the lack of assessments on the environmental and public health impact of the findings.

Future research should expand the profiling of resistance genes and mobile genetic elements to comprehensively understand the AMR landscape. Quantitative risk assessments of environmental and public health impacts and intervention studies to evaluate improved wastewater management practices and antimicrobial use policies are essential. Incorporating advanced statistical analyses will also strengthen the validation and predictive value of findings.

This study provides a critical foundation for understanding the molecular characteristics of ESBL-producing *E. coli* in East Java. It offers actionable insights for developing interventions to mitigate AMR and protect public health.

## DATA AVAILABILITY

All data supporting the findings of this study are available within the manuscript.

## AUTHORS’ CONTRIBUTIONS

FNAEPD: Conceptualized and designed the study and drafted manuscript. SMY: Data interpretation. MHE: Conceptualized and designed the study and edited the manuscript. HP and WT: Conceptualized and designed the study. ENU: Data interpretation and finalized the manuscript. RS: Interpreted the data and edited the manuscript. MAAS: Performed the laboratory procedures and collected samples. All authors have read and approved the final manuscript.
